# Biological Characteristics and Genomic Analysis of *Acinetobacter nosocomialis* Lytic Phage XC1

**DOI:** 10.3390/cimb47050335

**Published:** 2025-05-07

**Authors:** Chuhan Wang, Shuchuan Zhao, Hailin Jiang, Hongyan Shi, Jinghua Li, Chunyan Zhao, Honglan Huang

**Affiliations:** Department of Pathogen Biology, College of Basic Medical Science, Jilin University, Changchun 130021, China; chwang24@mails.jlu.edu.cn (C.W.); zhaoshuchuanzzu@gmail.com (S.Z.); jianghl23@mails.jlu.edu.cn (H.J.); hyshi@jlu.edu.cn (H.S.); ljh@jlu.edu.cn (J.L.); zhaocy@jlu.edu.cn (C.Z.)

**Keywords:** *Acinetobacter nosocomialis*, phage genome, phage therapy

## Abstract

This study aims to isolate and characterize the lytic phage XC1 targeting *Acinetobacter nosocomialis* and systematically analyze its biological properties and genomic structure, providing theoretical support for developing novel treatments against antibiotic-resistant infections. Phage XC1 was isolated and purified from lake water. Its morphology, optimal multiplicity of infection (MOI), thermal stability, and pH tolerance were analyzed. Genomic sequencing and functional annotation were performed to identify its lysis-associated genes. Phage XC1 demonstrated a short latent period (20 min) and high burst size (310 plaque-forming units per cell, PFU/cell). It remained stable under temperatures of 50–60 °C and at pH 7, indicating good environmental stability. Genomic analysis revealed a 45,324 bp genome with a GC content of 38.21%, including 84 open reading frames (ORFs), without any lysogenic, virulence, or antibiotic-resistance genes, confirming its safety. Average Nucleotide Identity (ANI) analysis shows that the ANI values between phage XC1 and other phages range from 80% to 95%. As the ANI value between strains of the same species is typically ≥95%, this suggests that phage XC1 may be a previously undiscovered new phage. Classified within the genus *Obolenskvirus* (class Caudoviricetes), phage XC1 is a virulent bacteriophage with rapid lytic activity and extreme environmental tolerance. Its therapeutic potential against multidrug-resistant infections, either as a monotherapy or in synergy with antibiotics, warrants further investigation.

## 1. Introduction

*A. nosocomialis* is a gram-negative, coccobacillus bacterium classified within the phylum γ-Proteobacteria. It is a prominent member of the *Acinetobacter calcoaceticus–A. baumannii (ACB) complex*, which has emerged as a major group of pathogens responsible for hospital-acquired infections in recent years. Within this complex, *A. nosocomialis* ranks second in terms of detection frequency, accounting for 21% of cases, following *Acinetobacter baumannii* at 63%. This bacterium is ubiquitously present in healthcare settings, particularly on the surfaces of medical devices such as intravenous catheters and ventilators, as well as non-medical surfaces, including door handles and bedrails [1,2]. *A. nosocomialis* is implicated in a wide range of serious infections, including pneumonia, meningitis, urinary tract infections, bacteremia, and sepsis. These infections not only pose significant threats to patient health but also substantially increase healthcare costs. Consequently, *A. nosocomialis* has become an increasingly recognized opportunistic pathogen that warrants further attention [3,4].

*A. nosocomialis* exhibits a notable ability to form biofilms, and recent studies have reported the identification of a strain capable of simultaneously forming biofilms and exhibiting resistance to multiple classes of antibiotics, including *cephalosporins*, *β-lactams*, *carbapenems*, *fluoroquinolones*, and *aminoglycosides*. Given the escalating resistance of *A. nosocomialis* to conventional antibiotics, there is an urgent need for alternative therapeutic strategies to manage infections caused by this pathogen [5,6]. In recent years, phage therapy has garnered significant attention as a potential antimicrobial treatment, offering a promising alternative to traditional antibiotics. In this study, bacteriophages capable of lysing *A. nosocomialis* were isolated from lake water. The biological properties and optimal conditions for phage stability were thoroughly investigated. Whole-genome sequencing of the isolated phage was performed to characterize its genomic structure, predict functional genomic regions, and analyze the phage lysis gene cassettes [7,8]. Furthermore, the sequencing data not only elucidated the phage’s genomic architecture but also contributed to expanding the phage genome database.

Finally, through whole-genome comparisons, we aim to elucidate the relationships and distinctions between this phage and others in its class, providing a theoretical foundation for the future application of phage therapy in the treatment of bacterial infections.

## 2. Materials and Methods

### 2.1. Materials

#### 2.1.1. Strain and Sewage

The *A. nosocomialis* clinical strain was provided by the Department of Pathogenic Biology, School of Basic Medical Sciences, Jilin University. Bacteriophage XC1 was isolated from lake water.

#### 2.1.2. Reagents and Instruments

Nutrient agar (NA) and nutrient broth (NB) were obtained from Qingdao Haibo Technology Co., Ltd. (Qingdao, China). Suspension Medium (SM) buffer was purchased from Wuhan Karnos Science and Technology Co., Ltd. (Wuhan, China). The centrifuge used in this study was from Thermo Fisher Scientific (Waltham, MA, USA), and the constant temperature incubator was supplied by Li-Chen Science and Technology Co., Ltd. (Tainan, Taiwan). The fully automated rapid microbial mass spectrometry detection system (MALDI-TOF) was obtained from bioMérieux (Shanghai) Diagnostic Technology Co. (Shanghai, China).

### 2.2. Methods

#### 2.2.1. Preliminary Isolation and Acquisition of XC1 Phage

The *Acinetobacter* sp. strain was activated by inoculating it into a 10 mL centrifuge tube containing 5 mL of nutrient broth (NB) using an inoculation loop. The culture was incubated on a constant temperature shaker at 37 °C, 160 rpm until it reached the logarithmic growth phase.

Approximately 500 mL of lake water was collected and supplemented with 3 g of CaCl_2_. The mixture was allowed to stand for 30 min and then centrifuged at 4000 rpm (2500× *g*) for 10 min. The supernatant was collected and mixed with an equal volume of nutrient broth, followed by the addition of 5 mL of the activated *A. nosocomialis* suspension. This mixture was incubated overnight at 37 °C. The next day, the culture was centrifuged again at 4000 rpm (2500× *g*) for 10 min, filtered through a 0.45 μm filter to obtain the phage suspension, and stored at 4 °C.

To confirm phage activity, 100 μL of the *A. nosocomialis* suspension in its logarithmic growth phase was added to a test tube, followed by 5 mL of semi-solid medium at approximately 50 °C. After solidification, 2–3 drops of the initial phage extract were added to the surface of the medium. The plate was allowed to dry and then incubated at 37 °C for 12 h. The appearance of phage plaques at the center of the plate was observed.

#### 2.2.2. Isolation and Purification of Phage and Determination of Titer

A single phage plaque was picked and added to a host bacterial suspension, which was then incubated for 8 h. Afterward, the culture was centrifuged at 4000 rpm (2500× *g*) for 10 min, and the resulting supernatant was filtered through a 0.45 μm filter to obtain the initial phage purification solution.

To purify the phage further, 100 μL of the phage solution was serially diluted, mixed with 100 μL of host bacterial suspension, and added to 5 mL of semi-solid medium at approximately 50 °C. This mixture was poured onto a solid agar plate, allowed to solidify, and then incubated overnight at 37 °C. The phage plaques were picked, and the process was repeated 7–10 times until uniform plaque sizes were observed on the agar plates. After the final purification step, the number of plaques at each dilution was counted and the phage titer calculated using the formula: PFU/mL = number of plaques × dilution factor × 10.

#### 2.2.3. Determination of Optimal Phage Infection Multiplicity (MOI)

The phage solution with a known titer was serially diluted, and the host *Acinetobacter* sp. solution was adjusted to a concentration of 1 × 10^6^ colony-forming units per milliliter (CFU/mL). Equal volumes of the phage and bacterial suspensions were mixed at various ratios of phage titer to host bacteria concentration (100, 10, 1, 0.1, 0.01, 0.001, 0.0001) and incubated at 37 °C, 160 rpm for 4 h. After incubation, the mixture was filtered through a 0.45 μm filter, and the phage titer under each condition was determined using the double-layer plate method. The ratio yielding the highest phage titer was selected as the optimal MOI. The experiment was repeated four times.

#### 2.2.4. Determination of Phage One-Step Growth Curve

The phage solution was mixed with the *A. nosocomialis* suspension according to the optimal MOI to form a 1 mL system, which was incubated in a 37 °C water bath for 15 min. The mixture was then centrifuged at 10,000 rpm for 2 min. The supernatant was discarded, and the precipitate was re-suspended in NB. This was added to a conical flask containing 15 mL of NB, and the mixture was incubated in a constant temperature shaker at 37 °C, 160 rpm. At intervals of 20 min, 1 mL samples were taken and filtered through a 0.45 μm filter. Phage titers were determined using the double-layer plate method, and the phage growth curve was plotted based on the highest titer observed. The experiment was repeated four times.

#### 2.2.5. Determination of Phage Thermal Stability and pH Stability

(1) Thermal Stability of Phage

A phage solution with a titer of 1 × 10^8^ PFU/mL was divided into four equal 1 mL aliquots, each placed in a constant temperature water bath at 50 °C, 60 °C, 70 °C, or 80 °C. Every 10 min during the 0–60 min period, 100 μL samples were taken, which were serially diluted and mixed with the *A. nosocomialis* solution in its logarithmic growth phase. Phage titers were then determined using the double-layer plate method. This experiment was repeated four times.

(2) pH Stability of Phage

The pH of SM buffer was adjusted using concentrated sulfuric acid and sodium hydroxide, and pH was calibrated using a pH meter. A 990 μL aliquot of SM buffer at varying pH levels was mixed with 10 μL of the phage solution (1 × 10^11^ PFU/mL) and incubated in a 37 °C water bath for 15 min. After incubation, 100 μL of the mixture was serially diluted, and the phage titer was determined using the method described above. The experiment was repeated four times.

#### 2.2.6. Observation of Phage Morphology by Transmission Electron Microscopy

Phage particles were extracted using the PEG/NaCl precipitation method and resuspended in SM buffer for storage at 4 °C. To observe phage morphology, a 20 μL sample of the phage suspension was mixed with an equal volume of 0.5% glutaraldehyde and dropped onto a copper grid. After 15 min, the phage particles were negatively stained with 2% phosphotungstic acid for 1–2 min. Excess liquid was removed with filter paper, and phage morphology was observed using transmission electron microscopy at 80 kV.

#### 2.2.7. DNA Extraction and Whole-Genome Sequencing Analysis of XC1 Phage

To extract the DNA of the XC1 phage, 500 μL of phage concentrate was mixed with 2.5 μL of DNase1 (1 mg/mL) and 0.5 μL of RNaseA (1 mg/mL), and the mixture was incubated in a water bath at 37 °C for 1 h. Then, 25 μL of EDTA was added, followed by 25 μL of Proteinase K and 25 μL of SDS. The mixture was incubated in a water bath at 56 °C for 1 h. Phage DNA was then extracted using phenol-chloroform-isoamyl alcohol (25:24:1, *v*/*v*/*v*).

To this, 1 mL of pre-cooled 70% ethanol was added, followed by centrifugation at 4 °C and 10,000 rpm for 5 min. The supernatant was discarded, and the precipitate was air-dried before being dissolved in 20–50 μL of TE buffer. The DNA concentration was measured using a spectrophotometer, and the 260/280 ratio was maintained between 1.8 and 2.0. The phage DNA solution was stored at −20 °C for further analysis.

The DNA sample (above 50 μg) was sent to Hangzhou Lianchuan Biotechnology Co. (Hangzhou, China) for sequencing and genome structure analysis. Libraries were constructed using the Illumina TruSeq™ Nano DNA Sample Prep Kit (Illumina, Inc., San Diego, CA, USA). The optimized sequences were assembled de novo using ABySS version 2.3.5 with multiple k-mer parameters to obtain the optimal assembly. Subsequently, GapCloser version 1.12-r6 was employed to perform local gap filling and base correction on the assembled sequences.

#### 2.2.8. XC1 Phage Gene Function Prediction and Annotation

The open reading frames (ORFs) of the XC1 phage genome were predicted using the GeneMarkS version 4.32 online tool. The gene function was annotated by searching the NCBI (https://www.ncbi.nlm.nih.gov/, accessed on 15 March 2024), PHASTER (http://phaster.ca/, accessed on 15 March 2024), RAST (https://rast.nmpdr.org/, accessed on 15 March 2024), and UniProt (https://www.uniprot.org/, accessed on 15 March 2024) databases. Furthermore, PhageLeads (https://phageleads.dk/, accessed on 15 March 2024) was used to predict potential mild phage genes, virulence genes, and antibiotic-resistance genes in the XC1 genome. Non-coding RNA (ncRNA) sequences were detected using Infernal version 1.1.4 software. The genome was visualized using CGView version 2.0.

The structural features of the holin and endolysin proteins in the XC1 phage lysogen cassette were analyzed using Expasy to determine their isoelectric points, molecular weights, stability coefficients, aliphatic indices, and hydrophilicity. Their biological properties and secondary structures were predicted with SOPMA (https://npsa-prabi.ibcp.fr/cgi-bin/npsa_automat.pl?page=/NPSA/npsa_sopma.html, accessed on 15 April 2024), SignalP version 6.0, DeepTMHMM v1.0, and InterPro, while tertiary structures were modeled using SWISS-MODEL (https://swissmodel.expasy.org/, accessed on 15 April 2024) [9,10].

#### 2.2.9. Analysis of XC1 Phage Evolutionary Relationships

To examine the evolutionary relationship between XC1 phage and other *Acinetobacter* phages, BLAST version 2.13.0 comparisons were performed with whole-genome sequences and terminal large subunit (terminase large subunit) protein sequences in the NCBI database. The top 13 sequences with the highest homology (based on base or amino acid identity) were selected for phylogenetic tree construction using MEGA version 11.0.13. The Neighbor-Joining (N-J) method was employed with the bootstrap method set to 1000.

#### 2.2.10. Comparative Genomics Analysis of the XC1 Phage

Twenty-two phage genome sequences were selected (with four repeated sequences removed) to construct evolutionary relationships based on both whole-genome sequences and terminal large subunit protein sequences. The Average Nucleotide Identity (ANI) between these phages was calculated using the JSpeciesWS online platform (http://jspecies.ribohost.com/jspeciesws/, accessed on 15 April 2024), both by ANIb (based on BLAST) and ANIm (based on MUMmer). Covariance analysis was performed using ViPTree (https://www.genome.jp/viptree/, accessed on 15 April 2024), and covariance plots were generated using the sequences with the highest similarity to the XC1 phage based on both the ANIb and ANIm results.

## 3. Results

### 3.1. Isolation and Morphological Characterization of Phage XC1

**Figure 1:**
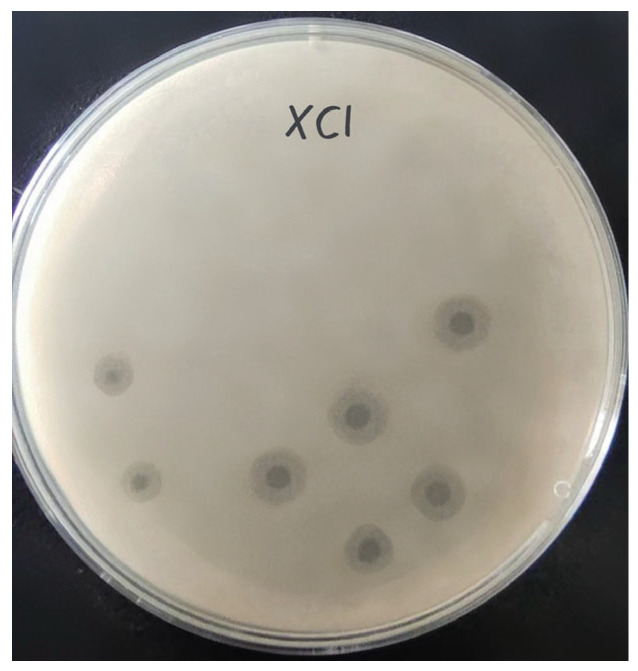
Plaques of phage XC1.

**Figure 2:**
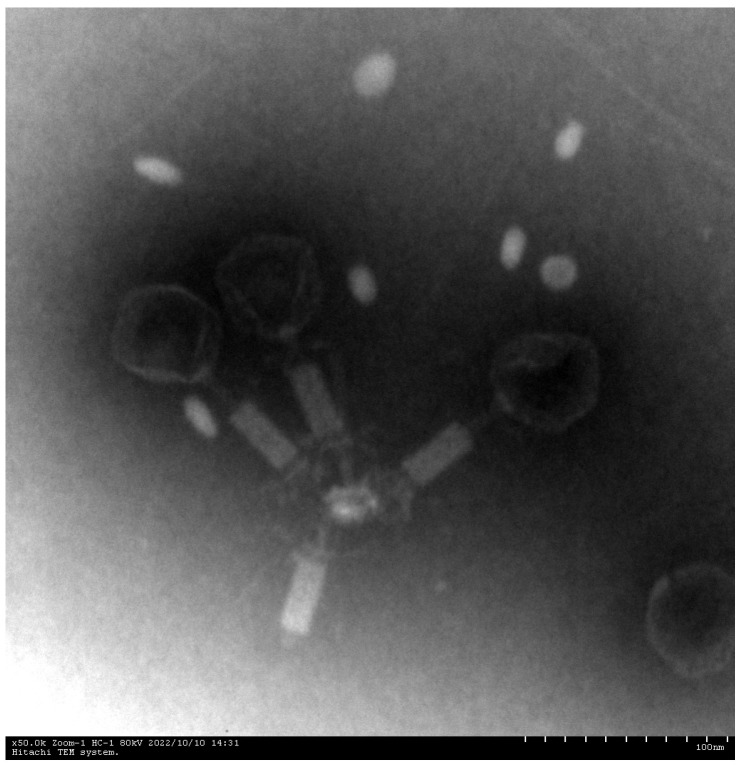
Electron micrographs of phage XC1 (100 nm scale).

Phage XC1 was isolated from lake water using *A. nosocomialis* as the host bacterium and subsequently purified through multiple rounds of filtration to obtain a clean phage preparation. This phage exhibits a distinct round, transparent plaque with a halo region measuring approximately 1–2 mm in diameter, as depicted in Figure 1. Morphologically, the XC1 phage (Figure 2) exhibits a three-dimensional head structure, with an average diameter of 61.4 ± 6.5 nm. The phage also features a contractile tail, with an approximate length of 64.5 ± 4.4 nm, classifying it as a *myovirus*.

### 3.2. Optimal Multiplicity of Infection (MOI) Assay for Phage XC1

**Figure 3:**
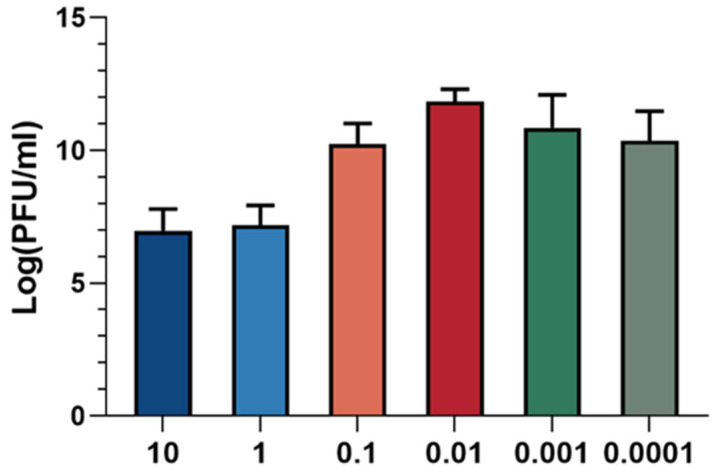
MOI of phage XC1.

The optimal multiplicity of infection (MOI) refers to the ratio of phage to host bacteria, which is critical for maximizing progeny production. The optimal MOI corresponds to the ratio at which the phage exhibits the highest efficiency in producing progeny. As illustrated in Figure 3, the phage titer was found to be highest when the ratio of XC1 phage to *A. nosocomialis* was 0.01, indicating that the optimal MOI for the XC1 phage is 0.01.

### 3.3. One-Step Growth Curve of Phage XC1

**Figure 4:**
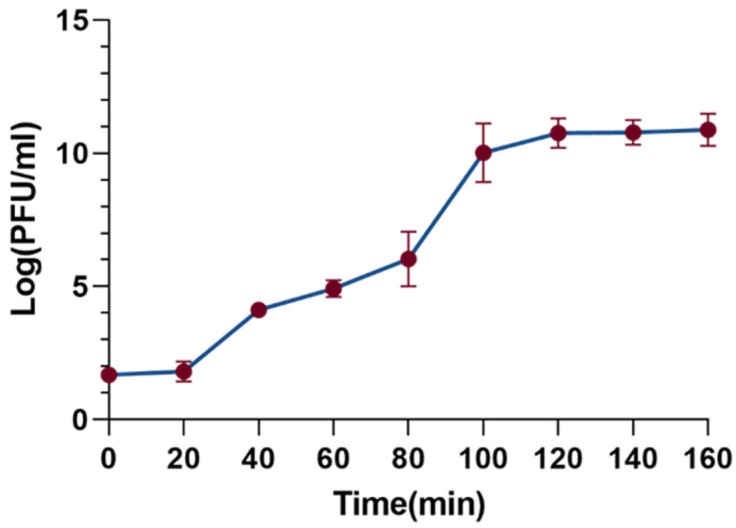
Step growth curve of phage xc1.

The one-step growth curve is a useful tool to characterize the phage’s latent period, burst period, and burst size. As shown in Figure 4, the latent period of the XC1 phage was determined to be 20 min, followed by a burst period of 80 min. During the burst phase of the infection cycle, the burst size of the XC1 phage reached 310 PFU per cell. The burst size was calculated by dividing the phage titer at the end of eruption (PFU/mL) by the initial number of infected bacterial cells (CFU/mL).

### 3.4. Determination of Phage Stability

#### 3.4.1. Thermal Stability of Phage XC1

**Figure 5:**
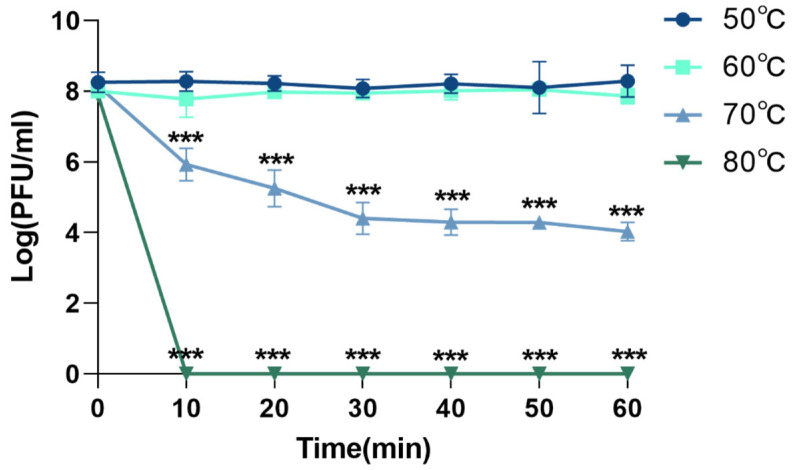
Temperature resistance of phage XC1 (comparison of phage activity with that at 50 °C, *** *p* < 0.001).

The thermal stability of phage XC1 was assessed by exposing the phage to temperatures of 50 °C, 60 °C, 70 °C, and 80 °C, as shown in Figure 5. At temperatures ranging from 50 °C to 60 °C, there was no significant change in the phage titer. However, when the temperature was raised to 70 °C, the titer of the XC1 phage decreased approximately 1000-fold within 30 min, with the rate of decrease slowing between 30 and 60 min. At 80 °C, the phage could no longer be detected, indicating that high temperatures significantly compromised the biological activity of the XC1 phage.

#### 3.4.2. pH Stability of Phage XC1

**Figure 6:**
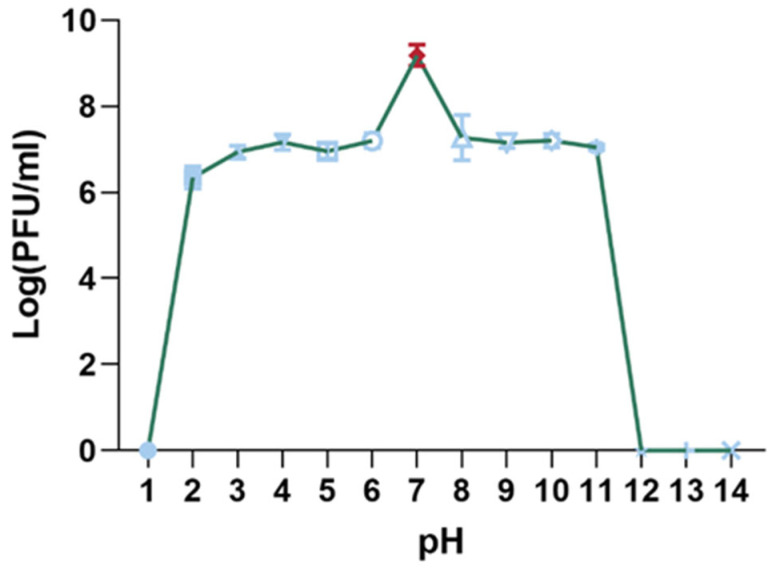
pH stability of phage XC1.

The pH stability of the XC1 phage was evaluated by incubating the phage in solutions with pH values ranging from 1 to 14 for 15 min, and subsequent titer measurements were taken, as shown in Figure 6. The highest phage titer was observed at pH 7, where the phage activity remained unaffected. Significant reductions in phage titer were noted between pH 2–6 and pH 8–11. The XC1 phage was barely detectable at pH 1 and at pH values greater than 11, suggesting that extreme acidic or alkaline conditions result in a considerable decrease or complete loss of phage activity.

### 3.5. Genome-Wide Analysis and Functional Annotation of Phage XC1

**Figure 7:**
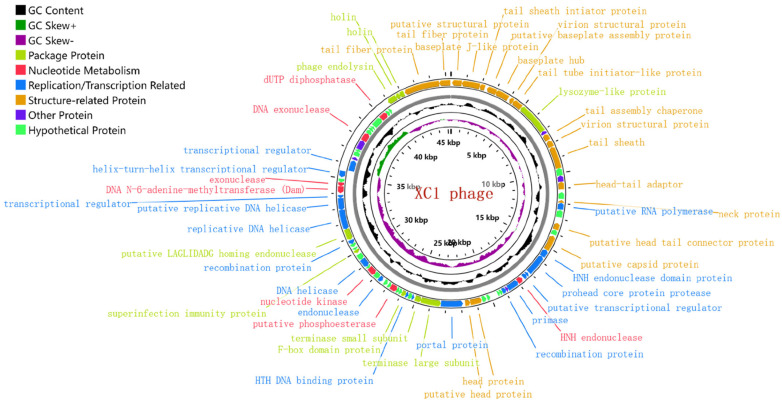
Genome-wide mapping of the XC1 phage. Note: The innermost loop is the genome size logo; the second loop is GC skew; the third loop is GC content; and the outer two turns are CDS on the positive and negative strands. The predicted 84 proteins were divided into six modules: packaging protein module, nucleotide metabolism module, replication/transcription-related protein module, structure-related protein module, other functional protein module, and putative protein module.

The genome of phage XC1 is composed of a circular DNA molecule (Figure 7) with a length of 45,324 base pairs and a GC content of 38.21%. Genome annotation using the GeneMarkS online tool predicted a total of 84 open reading frames (ORFs), with an average ORF length of 503 base pairs. These ORFs account for approximately 93.4% of the entire genome, with 13 genes located on the positive strand and 71 genes on the negative strand. The complete genome sequence of phage XC1 was deposited in GenBank under the accession number OQ547903.1.

The gene functions of phage XC1 were predicted using the NCBI, PHASTER (Phage Search Tool Enhanced Release), RAST (Rapid Annotation using Subsystem Technology), and UniProt databases, as summarized in Table 1. No proteins associated with lysogenic phages, such as transposases or integrases, were identified. Additionally, no genes related to mild phage traits, virulence, or antibiotic resistance were detected using the PhageLeads database. Furthermore, analysis with Infernal software did not reveal the presence of non-coding RNAs (ncRNAs). These findings suggest that the XC1 phage is genetically safe, lacking genes typically associated with pathogenicity or antibiotic resistance.

The innermost circle is the genome size identification; the second circle is GC skew value; the third circle is GC content; and the outer two circles are CDS on the positive and negative strands. The predicted 84 proteins were divided into six modules: packaging protein module, nucleotide metabolism module, replication/transcription-related protein module, structure-related protein module, other functional protein module, and putative protein module

### 3.6. Analysis of the Lysis System of Phage XC1

**Figure 8:**
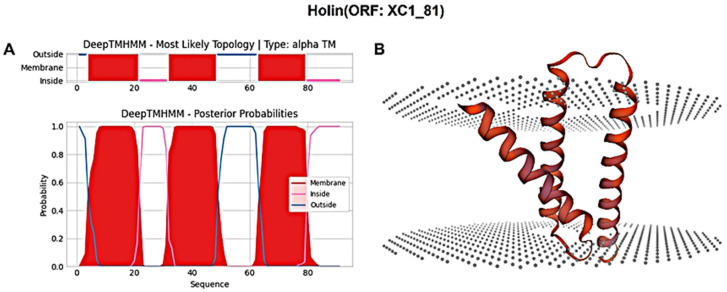
(**A**) Holin (ORF: XC1_81) transmembrane domain; (**B**) three-dimensional structure of holin (ORF: XC_81).

The holin protein (ORF: XC1_81) was analyzed for its amino acid composition, revealing a total of 91 amino acids; it had a molecular weight of approximately 10.6 kDa and an isoelectric point (PI) of 6.96. This protein contains seven negatively charged residues (Asp + Glu) and seven positively charged residues (Arg + Lys), with an instability coefficient of 21.36, indicating relative structural stability. The aliphatic index of the protein is 109.12, and the average hydrophilic (GRAVY) index is 0.635. Secondary structure prediction using SOPMA revealed the following composition: 47.25% α-helices, 20.88% extended strands, 13.19% β-turns, and 18.68% irregular coils. Signal peptide sequence prediction using SignalP-6.0 did not identify any signal peptide, suggesting that this holin protein is not secretory. Transmembrane domain prediction using DeepTMHMM identified three transmembrane structural domains, as shown in Figure 8A, classifying the protein as a class I perforin. The tertiary structure of the holin (ORF: XC1_81) protein was predicted using SWISS-MODEL, as shown in Figure 8B.

**Figure 9:**
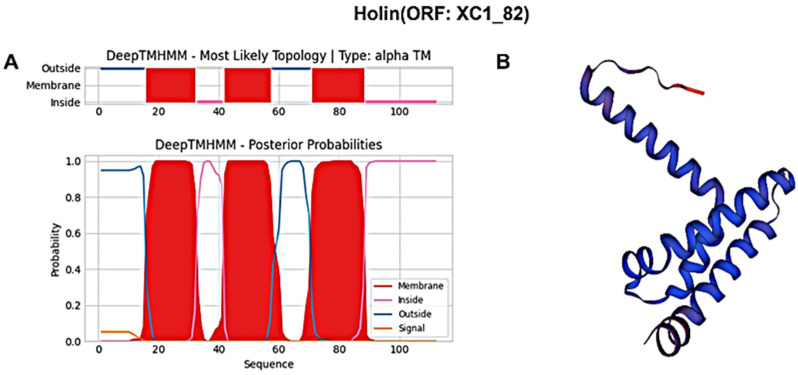
(**A**) Holin (ORF: XC1_82) transmembrane domain; (**B**) three-dimensional structure of holin (ORF: XC_82). Note: The X-axis represents the amino acid sequence position, and the Y-axis represents the predicted probability. Red areas represent transmembrane structural domains, and pink and blue lines represent intra- and extra-membrane structural domains, respectively.

Similarly, the holin protein (ORF: XC1_82) was analyzed, revealing a total of 112 amino acids; it had a molecular weight of approximately 11.9 kDa and an isoelectric point (PI) of 5.26. This protein contains 12 negatively charged residues (Asp + Glu) and 10 positively charged residues (Arg + Lys), with an instability coefficient of 23.37, indicating relatively stable structure. The aliphatic index is 120, and the average GRAVY index is 0.721. In secondary structure analysis, the protein is composed of 35.71% α-helices, 26.79% extended strands, 13.39% β-turns, and 24.11% irregular coils. No signal peptide sequence was detected using SignalP-6.0, indicating that this holin protein is also not secreted. DeepTMHMM predicted three transmembrane structural domains (Figure 9A), classifying this protein as a class I perforin. The tertiary structure of the holin (ORF: XC1_82) protein was also predicted using SWISS-MODEL, as shown in Figure 9B.

**Figure 10:**
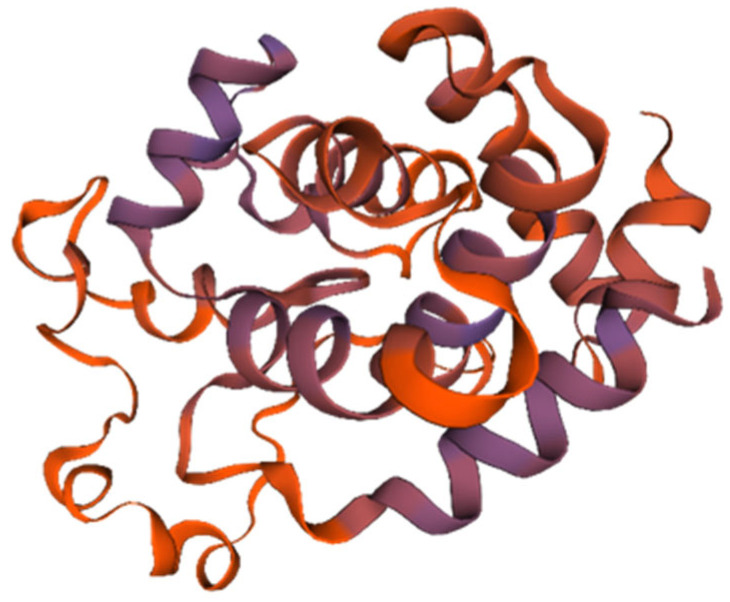
Three-dimensional structure of endolysin (ORF: XC1_80).

The endolysin protein (ORF: XC1_80) was analyzed, revealing a total of 202 amino acids, a molecular weight of approximately 23.2 kDa, and an isoelectric point (PI) of 9.42. This protein contains 22 negatively charged residues (Asp + Glu) and 31 positively charged residues (Arg + Lys), with an instability coefficient of 21.05, indicating structural stability. The aliphatic index is 74.41, and the average GRAVY index is −0.525. Secondary structure analysis revealed 50.5% α-helices, 10.29% extended strands, 4.46% β-turns, and 34.16% irregular coils. No signal peptide was predicted using SignalP-6.0, indicating that endolysin (ORF: XC1_80) is not a secreted protein. InterPro analysis revealed the presence of a lysozyme-like structural domain with glycoside hydrolase activity (amino acid sequence 88–139). The tertiary structure of the endolysin (ORF: XC1_80) protein was predicted using SWISS-MODEL, as shown in Figure 10.

### 3.7. Phylogenetic Evolutionary Analysis of Phage XC1

**Figure 11:**
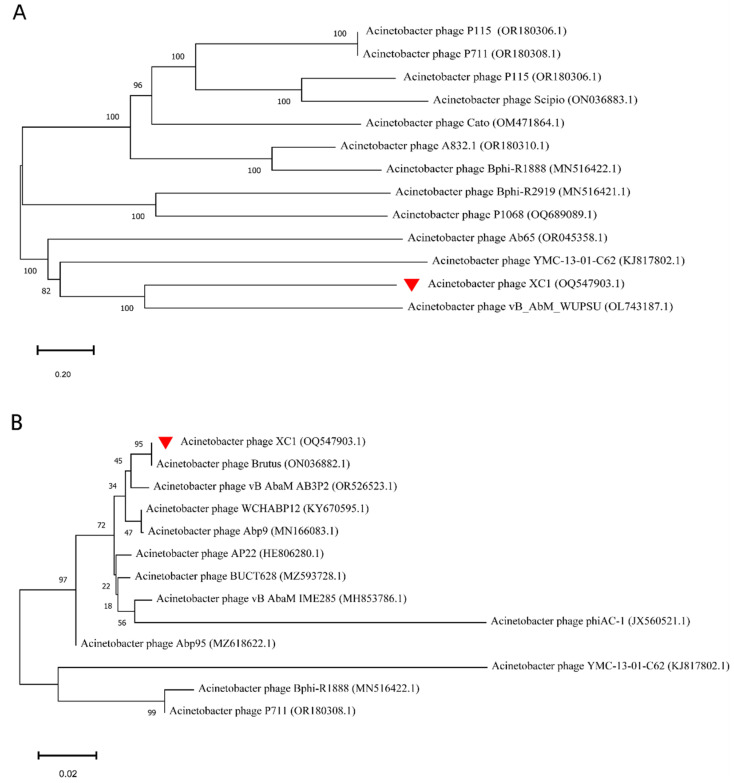
(**A**) Phylogenetic analysis of the complete genome of the XC1 phage. (**B**) Phylogenetic analysis of the terminase large subunit of XC1 phage.

The phylogenetic analysis of the whole-genome sequence of phage XC1, as shown in Figure 11A, revealed that the XC1 phage is closely related to *Acinetobacter* phage vB AbM WUPSU. Further evolutionary analysis of the terminal large subunit protein sequence (Figure 11B) demonstrated that the XC1 phage shares a closer phylogenetic relationship with *Acinetobacter* phage Brutus. Although the XC1 phage shows some molecular kinship with other phages, its genomic characteristics and differences in functional genes indicate that it possesses distinct functional potential, setting it apart from more conventional phages.

### 3.8. Comparative Genomics Analysis of Phage XC1

**Figure 12:**
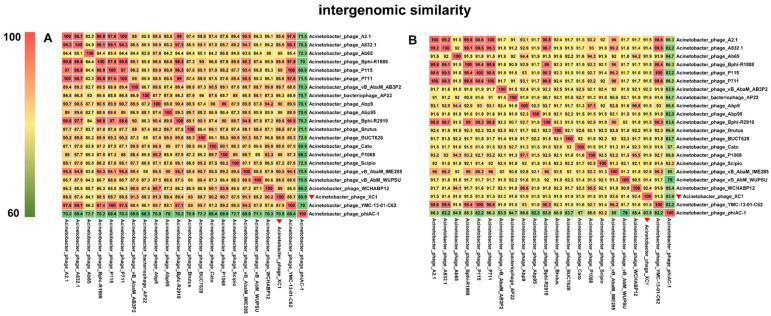
(**A**) Comparison of ANIb values between the XC1 phage and other phages. (**B**) Comparison of ANIm values between the XC1 phage and other phages.

Comparative genomic analysis of phage XC1 was conducted using the Average Nucleotide Identity (ANI) metric, comparing the XC1 phage with other phages. The ANI values based on the ANIb (Average Nucleotide Identity based on BLAST) principle are presented in Figure 12A. The ANI values between the XC1 phage and the other phages were found to be greater than 70% but less than 95%. Additionally, the ANI values obtained using the ANIm (Average Nucleotide Identity based on MUMmer) principle are shown in Figure 12B. These ANI values ranged from greater than 80% to less than 95%. Since phages belonging to the same species typically exhibit ANI values ≥ 95%, these findings suggest that the XC1 phage may represent a novel strain that has not been previously identified.

**Figure 13:**
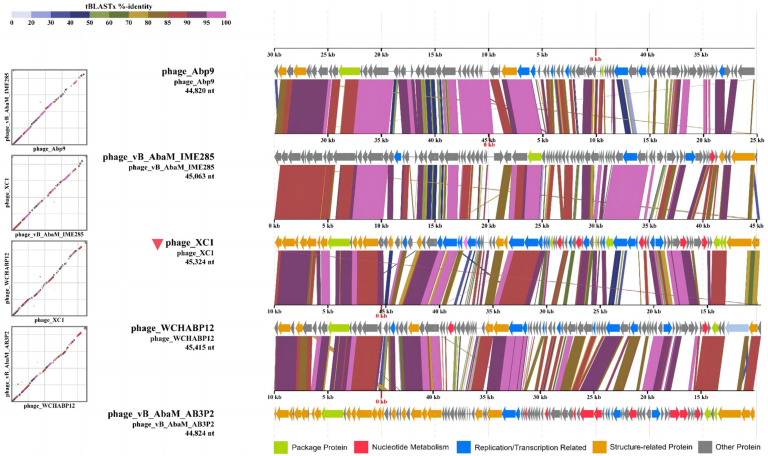
The homology comparison between the XC 1 phage and its proximal phages.

Figure 12A shows that the two sequences most similar to that of the XC1 phage were *Acinetobacter* phage Abp9 and *Acinetobacter* phage vB AbaM AB3P2, which had ANI values of 91.3%. From Figure 12B, the two sequences most similar to the XC1 phage were *Acinetobacter* phage vB AbaM IME285 and *Acinetobacter* phage WCHABP12, which had ANI values of 92.6% and 92.4%, respectively. These four sequences were selected for covariance analysis with the XC1 phage genome, as shown in Figure 13. The functional intervals of the genes in these phages were found to be similar, with most functional genes concentrated within the range of 85–100%. Despite the large differences in the arrangement of identical functional genes within the genomes, this suggests that the genome of the XC1 phage has likely undergone rearrangement.

## 4. Discussion

*A. nosocomialis*, a member of the *Acinetobacter calcoaceticus-Acinetobacter baumannii complex* (*ACB*), has been identified in various regions across multiple countries, with increasing recognition of its pathogenic potential. Its pathogenicity has become more widespread, with some areas reporting an even greater prevalence than *Acinetobacter baumannii* [11]. *A. nosocomialis* and other members of the ACB complex frequently contribute to mixed infections, which are common in hospital-acquired infections. These infections can lead to severe conditions such as pneumonia and bacteremia, particularly in immunocompromised or critically ill patients [12]. Members of the ACB complex are known for their strong biofilm-forming abilities, which significantly reduce the efficacy of therapeutic interventions. Additionally, the overuse and misuse of antibiotics have led to a rising number of resistant strains, with drug-resistant hospital-acquired *Acinetobacter* species being increasingly detected in clinical settings.

The global issue of antibiotic resistance has become increasingly severe. Based on the unique characteristics of bacteriophages and results from animal studies, phage therapy has demonstrated substantial potential as an alternative treatment approach. Some of the inherent limitations of phage therapy, such as the narrow host range, have been partially addressed through advances in genetic engineering technologies that enable receptor range expansion and the construction of engineered phages with reduced intrinsic toxicity. In addition, the research and development of phage-based products has progressed rapidly in several countries, particularly in Europe and the United States. Notably, the European Pharmacopoeia included phage active substances for human and veterinary use in 2021. The U.S. Food and Drug Administration (FDA) has also approved 20 bacteriophage-based products for applications in animal and food safety, among which seven are classified as investigational new drugs (INDs) for human use [13]. Phage therapy brings new hope for addressing the escalating problem of bacterial resistance, offering not only novel therapeutic strategies but also contributing to the reduction of antibiotic overuse and promoting environmental improvements.

In this study, a lytic phage, XC1, was isolated from lake water using *A. nosocomialis* as the host bacterium. The biological properties and whole-genome sequence of the phage were analyzed. The results showed that XC1 could form clear plaques on double-layer agar plates, producing a transparent halo ring. This suggests that the phage releases extracellular solvents, such as depolymerizing enzymes, following the lysis of host bacteria, which continue to degrade the surrounding uninfected bacteria, thereby facilitating the diffusion of phage particles within the bacterial population [14].

Electron microscopy revealed that the XC1 phage belongs to the *Obolenskvirus* genus within the Caudoviricetes class, which is characterized by a contractile tail, one of the most common and widely distributed morphologies for bacteriophages. This morphology is similar to that of the TCUAN1 phage, which also infects *Acinetobacter* species in hospital settings. Both phages form transparent plaques with halo rings, but XC1 has a shorter latent period (approximately 20 min) compared to TCUAN1, which has a latency period of about 30 min. Additionally, the XC1 phage has a higher burst size (310 PFU/cell) than the TCUAN1 phage (47 PFU/cell), suggesting that phages with shorter latency periods may replicate more rapidly and efficiently release progeny phages [15].

Host range analysis of the XC1 phage revealed that it was unable to lyse 12 strains of *Acinetobacter baumannii*, *Acinetobacter calcoaceticus*, and *Acinetobacter lophilus*. This may be due to the high specificity of the phage resulting in a narrow host spectrum. Alternatively, this limitation could be attributed to the insufficient variety of strains used in the experiment. The one-step growth curve indicated that the XC1 phage has a short latent period of just 20 min, followed by a burst period of 80 min. This rapid progression to the plateau phase suggests that the replication rate of this phage is relatively fast, allowing for the production of a large number of progeny phages in a short time.

Thermal stability tests showed that the activity of XC1 was notably affected at 70 °C, but the phage remained active until 80 °C. These results suggest that the XC1 phage is relatively heat-resistant and may be able to tolerate extreme environmental conditions.

The genome of the XC1 phage significantly differs from that of known phages, indicating that it is a novel phage. Functional gene predictions revealed no genes associated with lysogeny, supporting the hypothesis that XC1 is a virulent phage. Moreover, the presence of key proteins such as holin and endolysin—critical for bacterial lysis—was predicted, along with a lysozyme-like protein capable of biofilm degradation. Bacterial biofilms are known to increase bacterial resistance significantly, with biofilm-associated bacteria being up to 1000–5000 times more resistant to antibiotics than planktonic bacteria [16]. The potential ability of the XC1 phage to degrade biofilms offers a clear advantage for its application in hospital settings. These findings highlight the potential of XC1 for therapeutic applications.

With the advancement of phage-based therapies, the XC1 phage holds great potential as a standalone treatment or as part of phage cocktail therapies. Combining multiple phages could broaden the spectrum of treatment and overcome the host-specific limitations of individual phages [17,18]. Furthermore, the combination of phage therapy with antibiotics could enhance antimicrobial efficacy and reduce bacterial resistance, providing a rational approach to overcoming the limitations of traditional therapies. The stability and specificity of phages such as XC1 are key factors that support the future development of phage formulations and their clinical application [19].

## Figures and Tables

**Table 1:** Functional annotation of phage genome-encoded proteins.

ORF	Locus	Length	Function	Protein ID
XC1_01	−	627	putative structural protein	WFD61208.1
XC1_02	−	1185	baseplate J-like protein	WFD61209.1
XC1_03	−	354	tail sheath intiator protein	WFD61210.1
XC1_04	−	648	putative baseplate assembly protein	WFD61211.1
XC1_05	−	891	baseplate hub	WFD61212.1
XC1_06	−	279	virion structural protein	WFD61213.1
XC1_07	−	657	tail tube initiator-like protein	WFD61214.1
XC1_08	−	2091	lysozyme-like protein	WFD61215.1
XC1_09	−	213	putative tail-fiber/lysozyme protein	WFD61216.1
XC1_10	−	426	tail assembly chaperone	WFD61217.1
XC1_11	−	450	virion structural protein	WFD61218.1
XC1_12	−	1464	tail sheath	WFD61219.1
XC1_13	−	495	hypothetical protein	WFD61220.1
XC1_14	−	414	cysteine protease	WFD61221.1
XC1_15	−	504	head-tail adaptor	WFD61222.1
XC1_16	−	447	hypothetical protein	WFD61223.1
XC1_17	−	186	neck protein	WFD61224.1
XC1_18	−	438	putative RNA polymerase	WFD61225.1
XC1_19	−	477	hypothetical protein	WFD61226.1
XC1_20	−	453	putative head tail connector protein	WFD61227.1
XC1_21	−	354	hypothetical protein	WFD61228.1
XC1_22	−	1008	putative capsid protein	WFD61229.1
XC1_23	−	495	HNH endonuclease domain protein	WFD61230.1
XC1_24	−	1347	prohead core protein protease	WFD61231.1
XC1_25	−	168	primase	WFD61232.1
XC1_26	−	276	putative transcriptional regulator	WFD61233.1
XC1_27	−	363	HNH endonuclease	WFD61234.1
XC1_28	−	777	primase	WFD61235.1
XC1_29	−	150	putative membrane protein	WFD61236.1
XC1_30	−	282	recombination protein	WFD61237.1
XC1_31	−	183	hypothetical protein	WFD61238.1
XC1_32	−	171	hypothetical protein	WFD61239.1
XC1_33	−	231	hypothetical protein	WFD61240.1
XC1_34	−	279	hypothetical protein	WFD61241.1
XC1_35	−	771	head protein	WFD61242.1
XC1_36	−	345	putative head protein	WFD61243.1
XC1_37	−	1440	portal protein	WFD61244.1
XC1_38	−	1302	terminase large subunit	WFD61245.1
XC1_39	−	432	terminase small subunit	WFD61246.1
XC1_40	−	165	primase	WFD61247.1
XC1_41	−	243	hypothetical protein	WFD61248.1
XC1_42	−	201	HTH DNA binding protein	WFD61249.1
XC1_43	−	276	F-box domain protein	WFD61250.1
XC1_44	−	195	hypothetical protein	WFD61251.1
XC1_45	−	210	hypothetical protein	WFD61252.1
XC1_46	−	444	putative phosphoesterase	WFD61253.1
XC1_47	−	222	hypothetical protein	WFD61254.1
XC1_48	−	240	hypothetical protein	WFD61255.1
XC1_49	−	282	endonuclease	WFD61256.1
XC1_50	−	390	hypothetical protein	WFD61257.1
XC1_51	−	534	nucleotide kinase	WFD61258.1
XC1_52	−	165	hypothetical protein	WFD61259.1
XC1_53	−	576	DNA helicase	WFD61260.1
XC1_54	−	408	hypothetical protein	WFD61261.1
XC1_55	−	213	superinfection immunity protein	WFD61262.1
XC1_56	−	162	hypothetical protein	WFD61263.1
XC1_57	−	222	hypothetical protein	WFD61264.1
XC1_58	−	267	recombination protein	WFD61265.1
XC1_59	−	729	putative LAGLIDADG homing endonuclease	WFD61266.1
XC1_60	−	1314	replicative DNA helicase	WFD61267.1
XC1_61	−	774	putative replicative DNA helicase	WFD61268.1
XC1_62	−	198	transcriptional regulator	WFD61269.1
XC1_63	−	456	DNA N-6-adenine-methyltransferase	WFD61270.1
XC1_64	−	243	exonuclease	WFD61271.1
XC1_65	−	252	hypothetical protein	WFD61272.1
XC1_66	−	426	helix-turn-helix transcriptional regulator	WFD61273.1
XC1_67	+	762	transcriptional regulator	WFD61274.1
XC1_68	+	213	DUF551 domain-containing protein	WFD61275.1
XC1_69	+	336	hypothetical protein	WFD61276.1
XC1_70	+	183	hypothetical protein	WFD61277.1
XC1_71	+	681	ERF family protein	WFD61278.1
XC1_72	+	603	DNA exonuclease	WFD61279.1
XC1_73	+	294	hypothetical protein	WFD61280.1
XC1_74	+	195	hypothetical protein	WFD61281.1
XC1_75	+	162	hypothetical protein	WFD61282.1
XC1_76	+	723	hypothetical protein	WFD61283.1
XC1_77	+	540	dUTP diphosphatase	WFD61284.1
XC1_78	+	231	hypothetical protein	WFD61285.1
XC1_79	+	198	hypothetical protein	WFD61286.1
XC1_80	−	609	phage endolysin	WFD61287.1
XC1_81	−	276	holin	WFD61288.1
XC1_82	−	339	holin	WFD61289.1
XC1_83	−	2328	tail fiber protein	WFD61290.1
XC1_84	−	726	tail fiber protein	WFD61291.1

## Data Availability

The complete genome sequence of phage XC1 was deposited in GenBank under accession number [OQ547903.1]. The deposited data are publicly available as of the date of publication.
